# Regional characterization and the influence of mineralogy on the swelling of lime-treated expansive soils

**DOI:** 10.1371/journal.pone.0359564

**Published:** 2026-10-09

**Authors:** Abdullah A. Shaker, Muawia Dafalla

**Affiliations:** Research Chair in Expansive Soils, Civil Engineering Dep, College of Engineering, King Saud University, Riyadh, Saudi Arabia; Beijing University of Technology, CHINA

## Abstract

The expansive soils pose significant challenges in geotechnical engineering due to their tendency to swell when wet and shrink when dry. They present serious issues and challenges to light structures and pavements. Treatment using hydrated lime is one of the stabilization techniques that is widely used to improve the performance of these soils. This study aims to examine the behavior of volume change for three types of expansive clays from three different locations in Saudi Arabia that are classified as expansive. Experimental work covered lime-treated and untreated clay. The influence of lime content and curing period is monitored. Four lime contents: 2%, 4%, 6%, and 8% by weight of dry clay were investigated. All tested samples were investigated at three different curing periods: 1, 10, and 28 days. Laboratory investigation included determining pH values, conducting one-dimensional odometer tests, and measuring suction. To achieve a 90% reduction in the swell potential of expansive clays, it was found that Tabuk clay requires at least 2–3% lime addition, while Al-Qatif clay requires 3–4% lime addition. Al-Ghatt clay requires more than 4% to reduce the swelling potential by 90%. These differences are attributed to the mineralogy of the clay and the pozzolonic reactions as a result of adding hydrated lime.

## 1 Introduction

Expansive soils fluctuate significantly in volume (shrinkage or swelling) in response to variations in moisture content. This means they can swell when wet and shrink when dry, which can cause major problems for buildings and infrastructure. This type of soil includes a wide range of clays that differ in their geological origin, mineralogy, and, swelling characteristics.Several locations in the Kingdom of Saudi Arabia reported expansive clays in areas such as the eastern province, central region, and northern region [1–6]. Extensive literature on expansive soils has documented and highlighted extensive damage that necessitates expensive repairs and raised safety concerns [7–9]. There are many methods by which the challenges posed by expansive soils can be mitigated through a variety of techniques. Mechanical methods include soil blending, compaction-controlled replacement, and surcharge loading [10]. Chemical stabilization involves adding materials like fly ash, cement, and lime to improve the soil properties [11–13]. Alternative approaches using scrap tires, polymers and recycled materials have also been explored [10,14]. a Construction and demolition materials have also been investigated [15–20]. Each treatment method has its advantages and limitations in terms of efficiency, cost-effectiveness, and environmental impact [10,11,21]. While progress has been made in understanding and modifying expansive soils over the past 60 years, there is still a need for improved standards and optimized stabilization protocols [9,11,22]. Lime is a chemical compound that reacts with clay minerals to form new, stronger compounds. This process also reduces the plasticity of the soil, making it less prone to deformation [23–26]. The improvement in expansive soil structure achieved by lime addition is attributed to chemical and pozzolanic and cation exchanges [13,27–31]. Cation exchange is considered a rapid process that takes place immediately after lime is added. In general, the cation exchange plays a critical role in reducing the swelling potential of expansive soils by altering their surface properties and promoting flocculation and pozzolanic reactions [32,33]. In addition, the extent of cation exchange depends on several factors, including the type and amount of clay present, the type of lime used, and the soil’s pH value.

In contrast, the pozzolanic reactions occur over a longer period (days, weeks, or years) and contribute to the long-term stability of the stabilized expansive soils. This reaction plays a significant role in enhancing soil strength, reducing plasticity, and improving durability, contributing to the construction of safe and reliable structures in challenging ground conditions [29,34–36].

Expansive clays pose significant challenges in construction due to their volume change behavior. Research has shown that stabilization with hydrated lime can effectively mitigate these issues. Studies indicate that lime treatment reduces swelling potential and swelling pressure, improves consolidation characteristics, and enhances strength properties [9,33,37–42]. Alternative stabilizers, such as lignosulfonate and dolomitic limestone byproduct, have also shown promise in reducing the volume change and improving soil properties [41,42]. These stabilization techniques are particularly beneficial in semi-arid regions where expansive soil problems are more pronounced [39]. Despite the widespread use of lime as a chemical stabilizer for such problematic soils, the interactive effects of lime content and curing duration on the hydro-mechanical behavior—particularly in terms of volume change, compressibility, and suction—remain inadequately quantified across different soil mineralogies. Moreover, most previous studies have focused either on limited soil types or specific parameters without providing a comprehensive evaluation across multiple expansive clay sources. To address this research gap, this study investigates the behavioral transformation in volume change of three types of expansive clays stabilized with varying lime contents (2, 4, 6, and 8%) and subjected to different curing periods (1, 10, and 28 days). The study focuses on characterizing the effects of lime content and curing time on key parameters, including swelling potential, compressibility, and suction. Understanding these relationships provides critical insights into the effectiveness of lime stabilization for expansive soils and its implications for various engineering applications, including foundation design, pavement construction, and embankment stability.

Although the application of hydrated lime for soil stabilization is well-established, this study provides a novel regional characterization by conducting a direct, side-by-side comparative analysis of three Saudi Arabian expansive clays that differ geographically and mineralogically (Al-Ghatt, Al-Qatif, and Tabuk). Unlike earlier research that often focuses on a particular soil type or isolated factors, this work systematically evaluates the interactive effects of lime concentration and curing period across distinct main mineralogies. It combines conventional one-dimensional oedometer testing with high-precision suction measurements using chilled mirror dew point technology. By providing precise, optimal lime-dosage thresholds necessary to achieve a 90% reduction in swell potential based on provincial soil characteristics rather than a universal standard, this dual approach offers a more thorough diagnostic of the behavioral alteration of these soils.Studying the detailed impact of hydrated lime at variable concentrations and curing times for clays of different mineralogies will contribute to the development of reliable and sustainable solutions and the mitigation of problems associated with expansive soils.

## 2 Materials

### 2.1 Expansive soils

Expansive soils used in this study were collected from three areas known for their problematic subsurface ground in Saudi Arabia. Fig 1 presents the map of these areas and their locations within the Kingdom of Saudi Arabia. The approval for collecting field samples was granted by Al-Ghatt, Al-Qatif, and Tabuk Municipality following a request from King Saud University to enable a team from the Research Chair in Expansive Soils to conduct extensive research and surveys of expansive soils within each region. All testing works were conducted in the advanced unsaturated soil laboratory at the College of Engineering.

**Fig 1:**
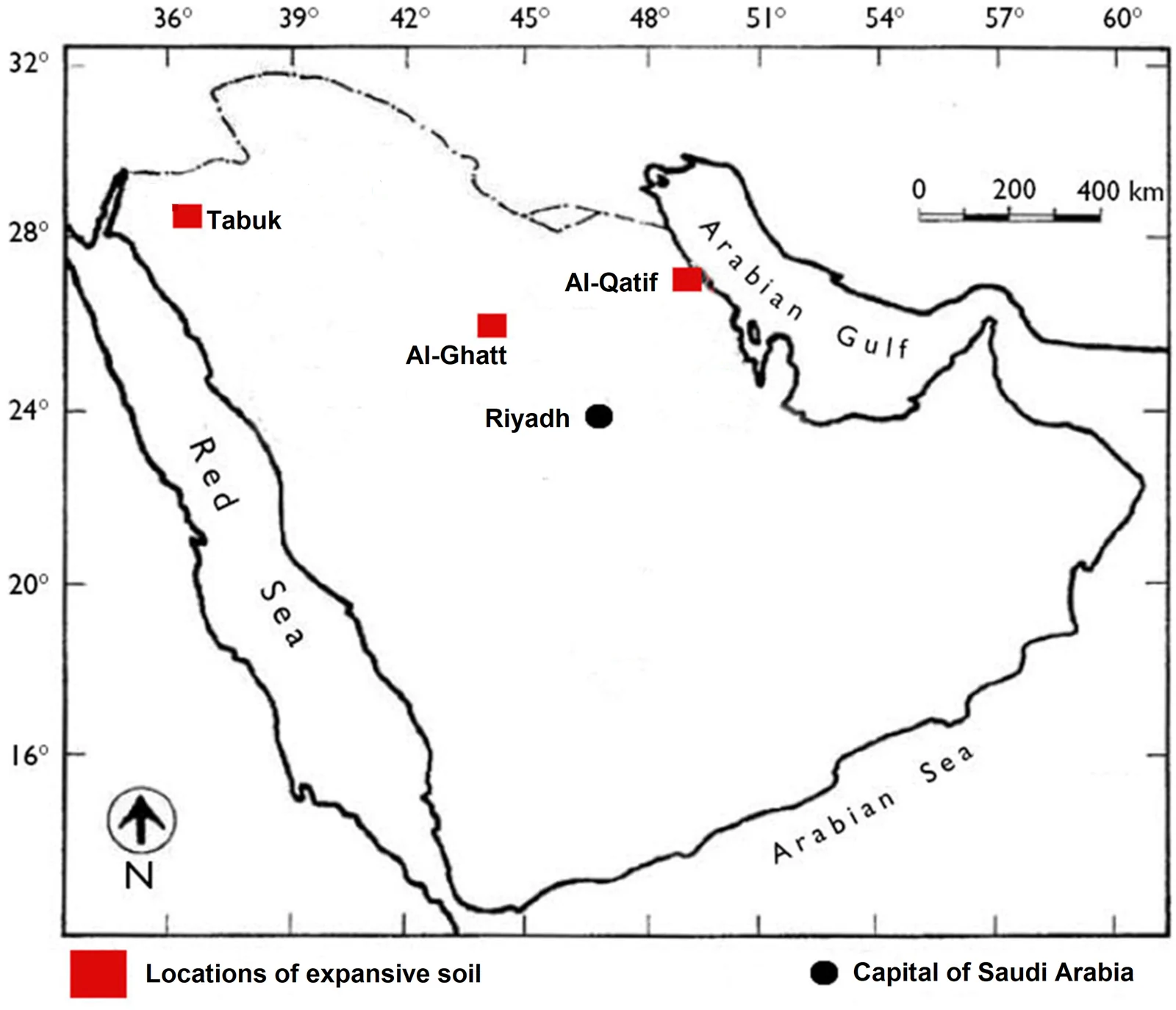
Area location of the studied clays (After [56]).

#### 2.1.1 Al-Ghatt expansive clay.

Al-Ghatt area is located in the Central Province of Saudi Arabia (referred herein as GEC). This type of problematic soil is believed to have formed from the weathering sediments deposited during the Jurassic period. The specific composition of these sediments likely included clays rich in illite, as identified through studies using X-ray diffraction. Dhowian et al. (1990) [43] were not able to identify smectite minerals in Al-Ghatt regions and claimed that the kaolinite and illite minerals observed in Al-Ghatt are of low to moderate expansion and cannot account for the expansive nature of the shale. Illite clays can exhibit some swelling behavior. This type of soil is classified as CL with low to moderate expansion potential [44–48]. Table 1 presents the geotechnical characterization of Al-Ghatt expansive clay.

**Table 1:** Geotechnical characteristics of expansive clays used.

Property	GEC	QEC	TEC
**Shrinkage limit (%)**	15	17.5	19.5
**Plastic limit (%)**	35	45	30
**Liquid limit (%)**	75	140	50
**Plasticity index (%)**	40	95	20
**Specific gravity**	2.86	2.76	2.81
**Swell index**	0.1–0.2	0.2–0.25	0.05–0.15
**Swelling pressure (kN/m**^**2**^)	100 - 400	500 - 800	150 - 250
**USCS classification**	CL	CH	CL
**%Finer than 200 μm**	87 - 90	97 - 98	91 - 94

#### 2.1.2 Al-Qatif expansive clay.

Al-Qatif area is located in the region along the Gulf coast, East of Saudi Arabia (referred herein as QEC). The soils of Al-Qatif are derived from the weathering of marl, calcareous limestone, and chert deposits from the Tertiary and Quaternary periods. The geological history, including glacial activity and fluctuating sea levels, has contributed to the formation of these expansive clays. The dominant clay minerals in Al-Qatif’s expansive clays are smectites, identified through X-ray diffraction analysis. This clay mineral has a layered structure that readily absorbs water, causing the soil to swell significantly in volume. Al-Qatif soil is classified as CH with high expansion potential [6,45,49–54]. Table 1 presents the geotechnical characterization of this clay.

#### 2.1.3 Tabuk expansive clay.

Tabuk area is located in the Northern Province of Saudi Arabia (referred herein as TEC). In this type of expansive soil, the source of the problem lies in the underlying Ordovician to Devonian shale formation. This sedimentary rock weathers into clay minerals, but unlike the dominant smectite in Al-Qatif, research suggests that kaolinite and mica are the most prevalent clay minerals in Tabuk’s expansive soils. While kaolinite can exhibit some shrinking and swelling behavior, it’s generally less expansive compared to smectite minerals. However, the presence of lesser amounts of illite, alongside the arid climate, can still contribute to problematic soil volume changes. This type of soil is classified as CL with low to moderate expansion potential [43,51]. Table 1 presents the geotechnical characterization of Tabuk expansive clay.

In general, Fig 2 indicates the soil mineralogy obtained using X-Ray diffractogram (XRD) for the expansive clay used in this study. Al-Ghatt clay (GEC) predominantly consists of kaolinite mineral, whereas Al-Qatif clay (QEC) has illite and palygorskite, while in Tabuk clay (TEC) kaolinite mineral is more dominant, Smectite minerals are known to induce considerable swelling upon interaction with water [55].

**Fig 2:**
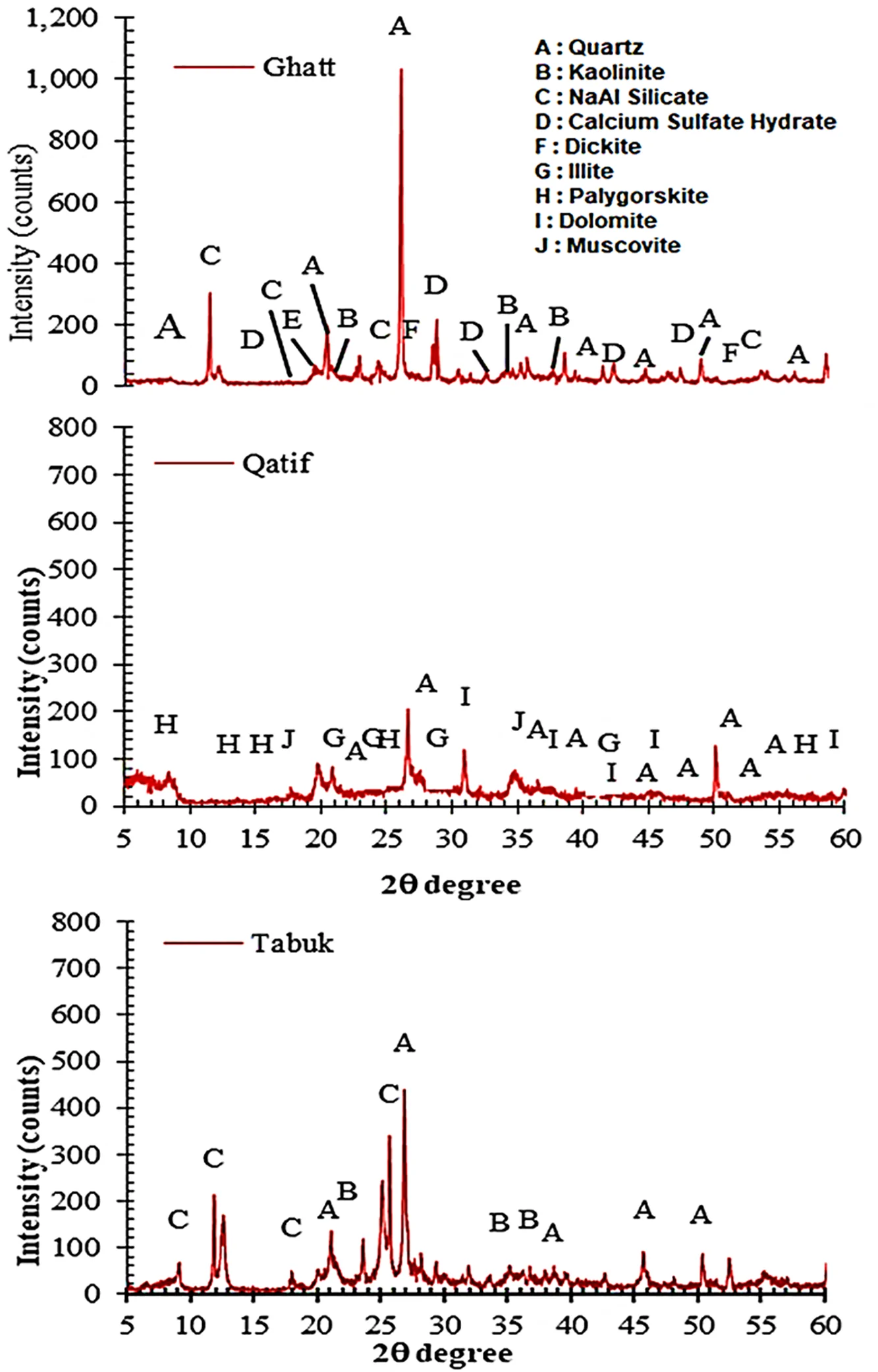
X-ray diffraction (XRD) analysis of the studied clays (After [47]).

#### 2.1.4 Lime.

Commercially available hydrated lime from Saudi Lime Co., Riyadh, KSA, was used in this study. Table 2 summarizes the manufacturer-provided properties of the hydrated lime. The lime contents considered in this study were 0% (untreated), 2%, 4%, 6%, and 8% by dry weight of clay.

**Table 2:** Main characteristics of hydrated lime used.

Property	Value (%)
Ca (OH)_2_	90 min
CaO ignited wt. basis	68.11
Combined water 550-660C^o^	21.89
MgO	1.0 max
CO_2_	2.0 max
Unhydrated oxide	0.2
SiO_2_ and insoluble	1.0 max
Fe2O_3_	0.05–0.1
Al2O_3_	0.2 max
Moisture 105C^o^	0.5 max
SO_3_	0.2 max

## 3 Methods

This section presents the procedure of the tests that were carried out for this research study. The natural expansive clays (GEC, QEC, and TEC) obtained from the field were air-dried, pulverized, and sieved using sieve No. 40 to form clay powders (Fig 3). All specimens tested were either untreated or lime-treated material. For all types of expansive clays, the range of lime content investigated included 0% (untreated), 2%, 4%, 6%, and 8% by dry weight of clay, which successfully covers the typical modification and stabilization optimums for all expansive clays. Required proportions of oven-dried lime and clay powder were hand mixed under dry conditions to obtain a homogeneous blend. Water content was then added and mixed thoroughly and stored in plastic bags for at least 16 hours in a humid area to allow time for the soil particles to hydrate as recommended by ASTM Standard D 698 [57]. The specimens used in this study were initially molded using static compaction to achieve the maximum dry density and the optimum moisture content (as per compaction characteristics). The treated samples were wrapped tightly in multiple layers of plastic cling-film, sealed inside airtight plastic bags to prevent moisture loss, and then placed in a controlled environmental chamber (Desiccator) to cure for 1, 10, and 28 days to evaluate the immediate modification, early strength development, and long-term pozzolanic cementation reactions, respectively. During curing, the relative humidity was maintained at 95% and at a temperature of 20°C ± 2°C, and in this condition, cementation bonds reach their maximum [44]. A digital calibrated pH meter was used to measure the pH value of the clay and clay lime mixtures.

**Fig 3:**
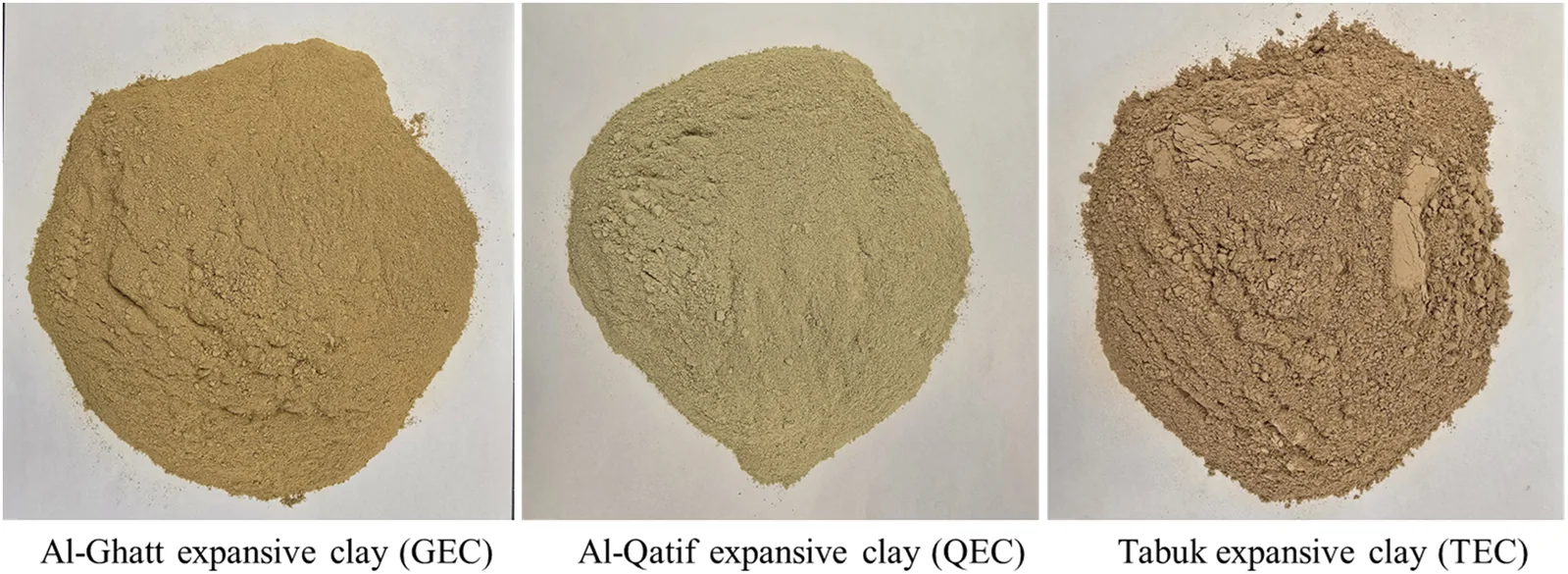
The studied natural expansive clays.

### 3.1 Compaction tests

The compaction tests were carried out in accordance with the standard Proctor compaction method (ASTM D698 - method A [57]). Dry mixing of the constituents was performed using hand to produce the most visible and texturally homogeneous mixtures. The dry expansive clay and dry lime were mixed and spread over a large pan and sprayed with distilled water using a spray bottle to achieve a uniform distribution of water. The mixtures were allowed to hydrate in plastic bags for at least 16 hours before compaction. Initial samples’ molding conditions considered in this study included samples prepared under optimum moisture content (OMC) conditions. The target dry densities (MDD) and moisture contents considered in this study are summarized in Table 3 for the expansive clay types selected.

**Table 3:** Initial dry density and moisture content for selected clays with various additives of lime.

Lime (%)	GEC		QEC		TEC	
	OMC, (%)	MDD (gm/cm^3^)	OMC, (%)	MDD (gm/cm^3^)	OMC, (%)	MDD (gm/cm^3^)
**0**	25.0	1.537	32.0	1.200	18.0	1.700
**2**	27.0	1.424	43.0	1.180	21.0	1.620
**4**	28.0	1.410	45.0	1.145	22.0	1.580
**6**	30.0	1.330	48.0	1.120	24.3	1.513
**8**	32.0	1.280	50.0	1.100	25.5	1.470

### 3.2 Suction tests

The suction values of clay specimens at the conditions considered in this study were evaluated using the WP4C chilled mirror dew point potentiometer, a device developed by Decagon, USA (Fig 4). The specimens for WP4C measurements were prepared using static compaction, utilizing a specially designed ring to produce specimens with a diameter of 36 mm and a height of 5 mm. To ensure measurement accuracy and counteract instrument drift, calibration was performed prior to each testing session using a standard salt solution (0.5 mol/kg from KCl), verifying that the readings fell within the acceptable tolerance ± 0.1 MPa. Each specimen was allowed to equilibrate inside the chamber until the temperature difference between the sample and the sensor stabilized (ΔT ≤ 0.1 C°), after which the total suction was recorded in continuous mode.

**Fig 4:**
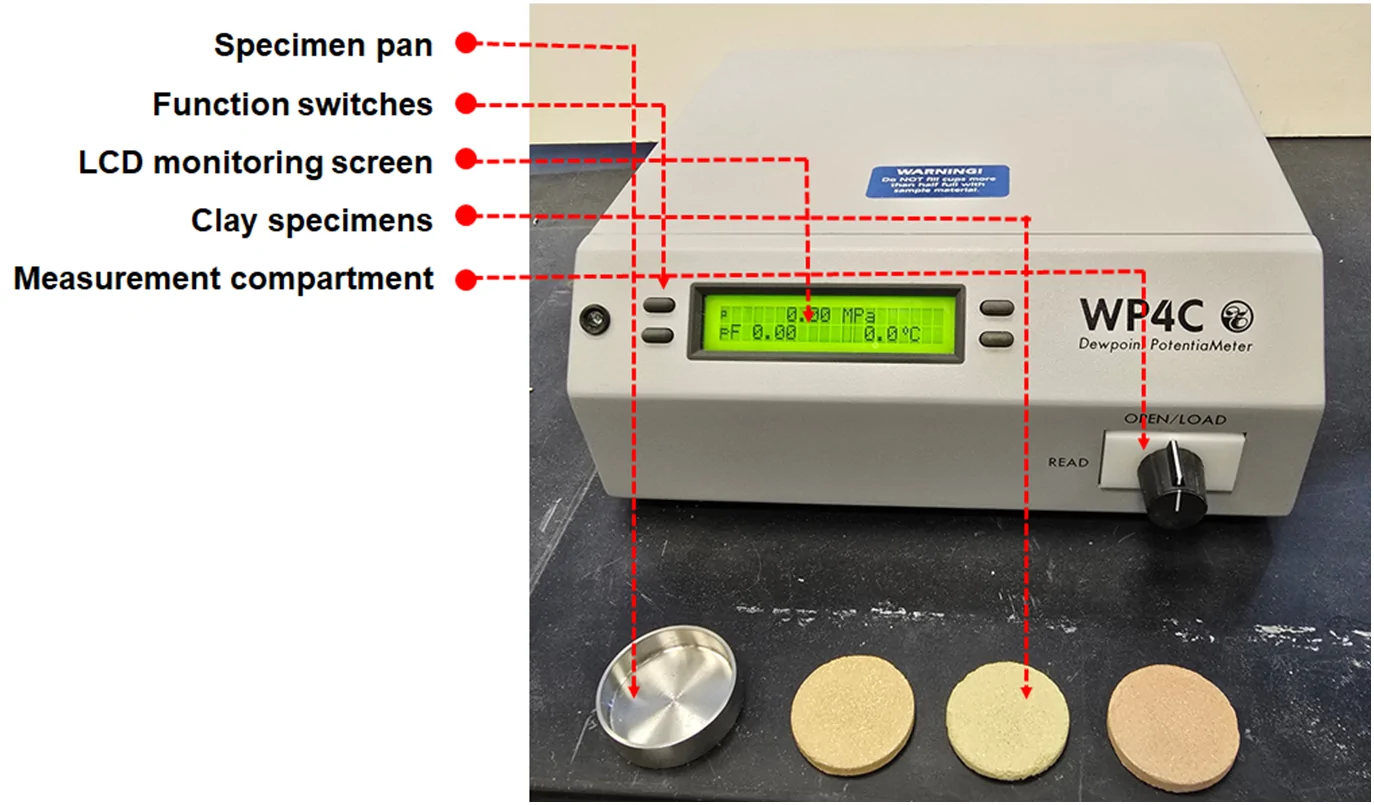
Photo of the WP4C chilled mirror dew point potentiometer used (after [58]).

### 3.3 Swelling and consolidation tests

Swelling and consolidation characteristics tests were performed using the one-dimensional oedometer device following the designations of ASTM D4546 [59]. A computer-controlled oedometer apparatus was used for the determination of volumetric parameters of the specimens. Remolded specimens were prepared by statically compacting in a stainless-steel ring of 63.5 mm in diameter and 20 mm in height (consolidometer ring) to the required density and water content. Silicone grease was applied to the inner surface of the ring to reduce side friction. Two porous stones and filter papers were placed at the top and bottom of the compacted specimen. Final specimen height was 16 mm, providing a 4 mm recess to allow for potential expansion of samples. A small surcharge load was applied (7 kPa), and then the specimen was inundated with distilled water and allowed to swell under this load, according to ASTM D4546 [59]. When no change in the vertical swell is noticed, the swell process is assumed to be achieved. The consolidation test was then performed by loading the specimen in increments following ASTM D 2435 [60] seven vertical stresses as of 14, 28, 56, 112, 224, 448, and 900 kPa were considered. After that, the applied stress on the specimen was reduced in decrements (i.e., 224 and 56 kPa) to obtain the unloading or rebound curve. Each increment of loading/unloading was continued for at least 24 h or until the vertical deformation vanished. Finally, the specimen was removed, and the water content was determined using gravimetric methods. The time required to complete the entire test is typically 8–10 days. The consolidation and unloading curves were plotted in terms of vertical stress (on log-scale) versus vertical strain. Using this curve, all volumetric parameters were evaluated, such as the swelling potential (SP), compression index (Cc), and swell index (Cs). A schematic representation of these curves is displayed in Fig 5, which includes the steps of the test and definitions for the key volumetric parameters.

**Fig 5:**
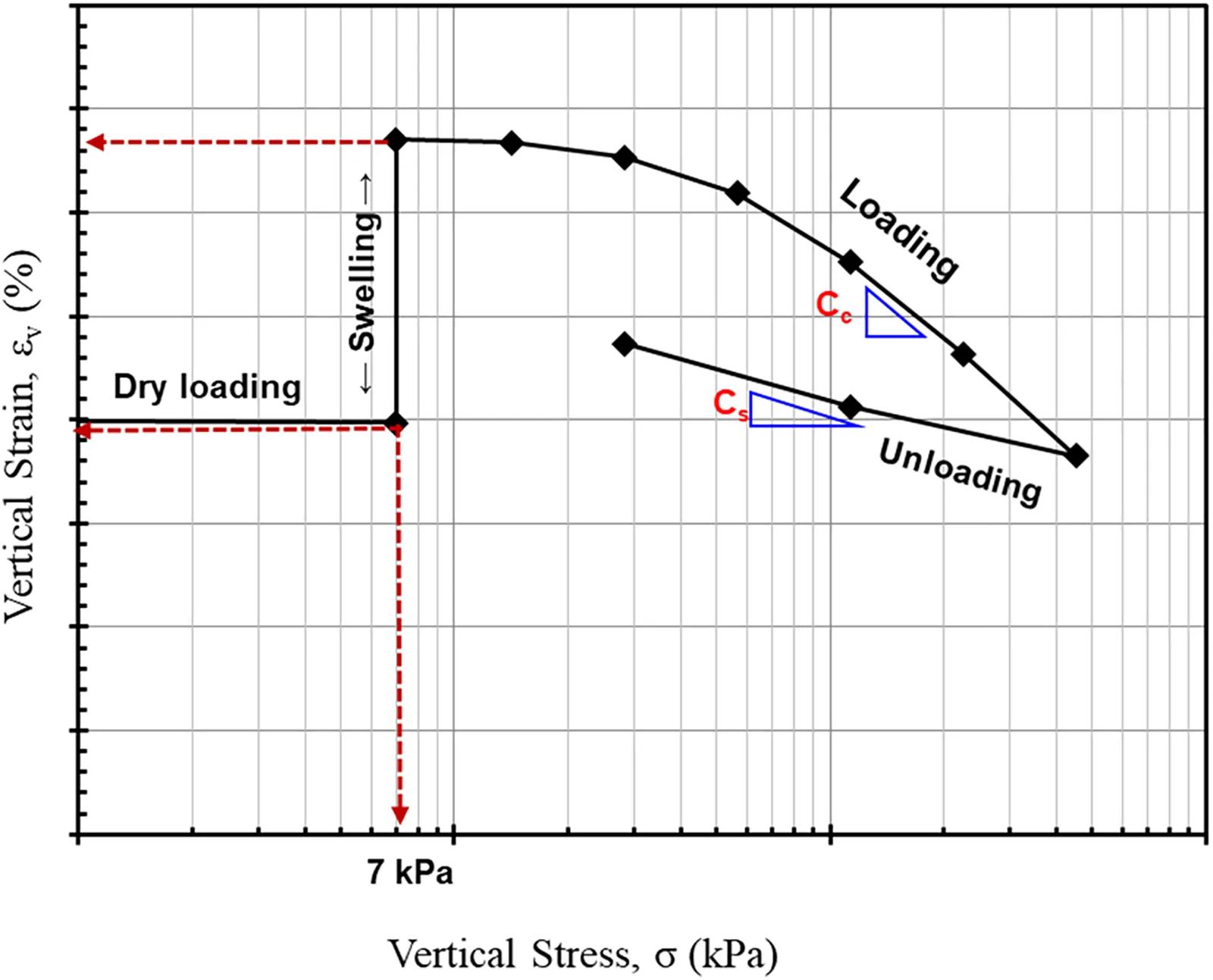
A typical consolidation curve illustrating the key volumetric parameters and basic steps of the test.

## 4 Results

### 4.1 pH Tests

The pH values obtained for the three clays tested with and without lime additives are presented in Fig 6. The values indicate a jump from low alkaline media (8) to alkalinity of between 12 and 13 for all hydrated lime additives of 2%, 4%, 6%, and 8%. Al-Qatif clays are of lower pH than the other clays in general.

**Fig 6:**
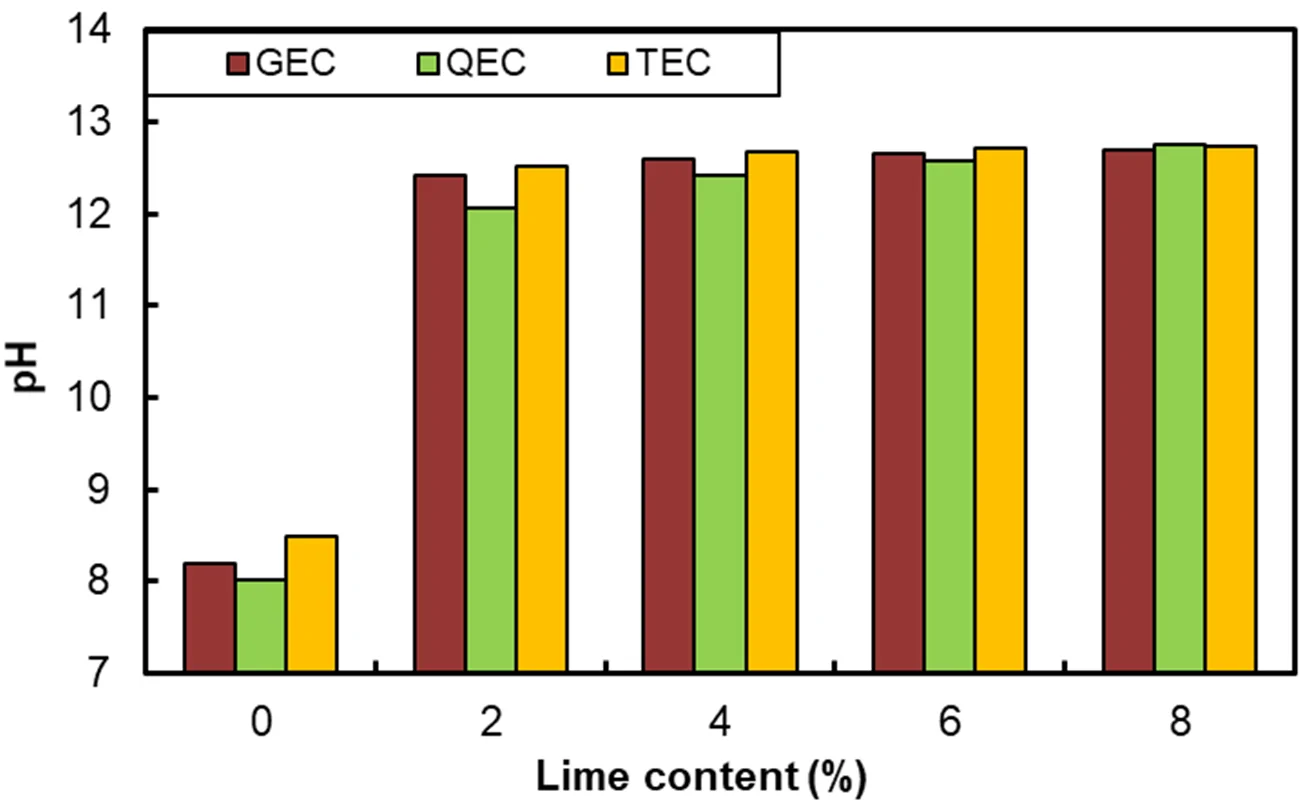
pH values for clays mixed with different proportions of lime.

### 4.2 Compaction data

Fig 7 presents the variation of optimum moisture content (OMC) and maximum dry density values with lime content that was considered in this study: 0%, 2%, 4%, 6% and 8% for all expansive clays. It is apparent from this figure that as the lime content increased, maximum unit weight decreased for all clays considered. However, as the lime content increased, the optimum moisture content increased for all clays tested.

**Fig 7:**
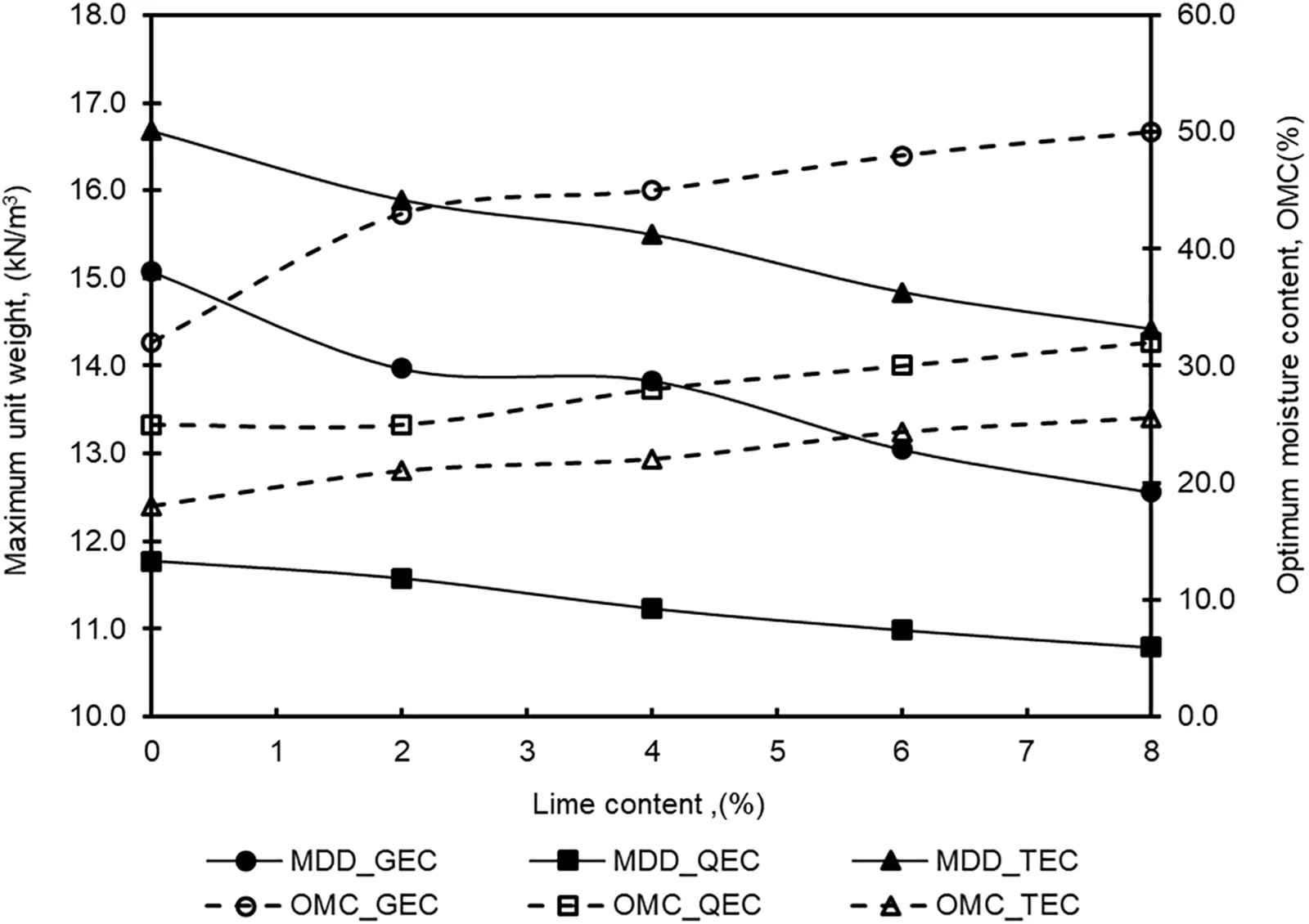
Maximum dry density and optimum moisture content values for clays mixed with different proportions of lime.

### 4.3 Swelling and consolidation

The vertical stress versus vertical strain for the untreated clay and clay treated with 2%, 4%, 6%, and 8% lime for Al-Ghatt clay are shown in Fig 8. For Al-Qatif clay the results are shown in Fig 9, and for Tabuk clay are shown in Fig 10. Fig 11 presents the swelling potential versus lime contents at all curing days for all types of clay (a) GEC, (b) QEC, and (c) TEC. Figs 12-13 presents the consolidation parameters represented by compression index (CC; Fig 12) and swell index (CS; Fig 13) versus lime contents at all curing days for all types of clay (a) GEC, (b) QEC, and (c) TEC.

**Fig 8:**
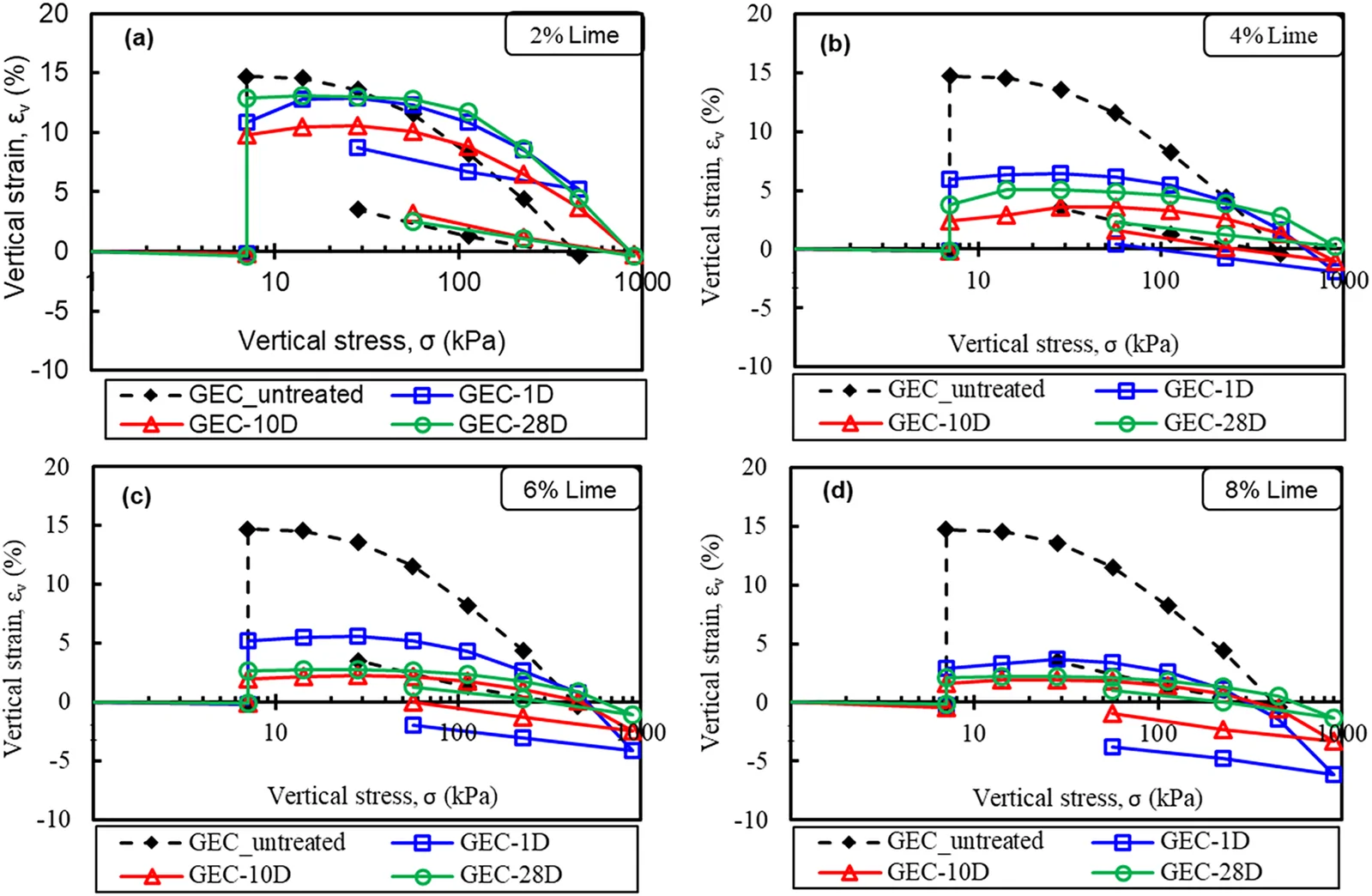
Consolidation curves of GEC under all curing days (a) at 2% lime, (b) at 4% lime, (c) at 6% lime and (d) at 8% lime.

**Fig 9:**
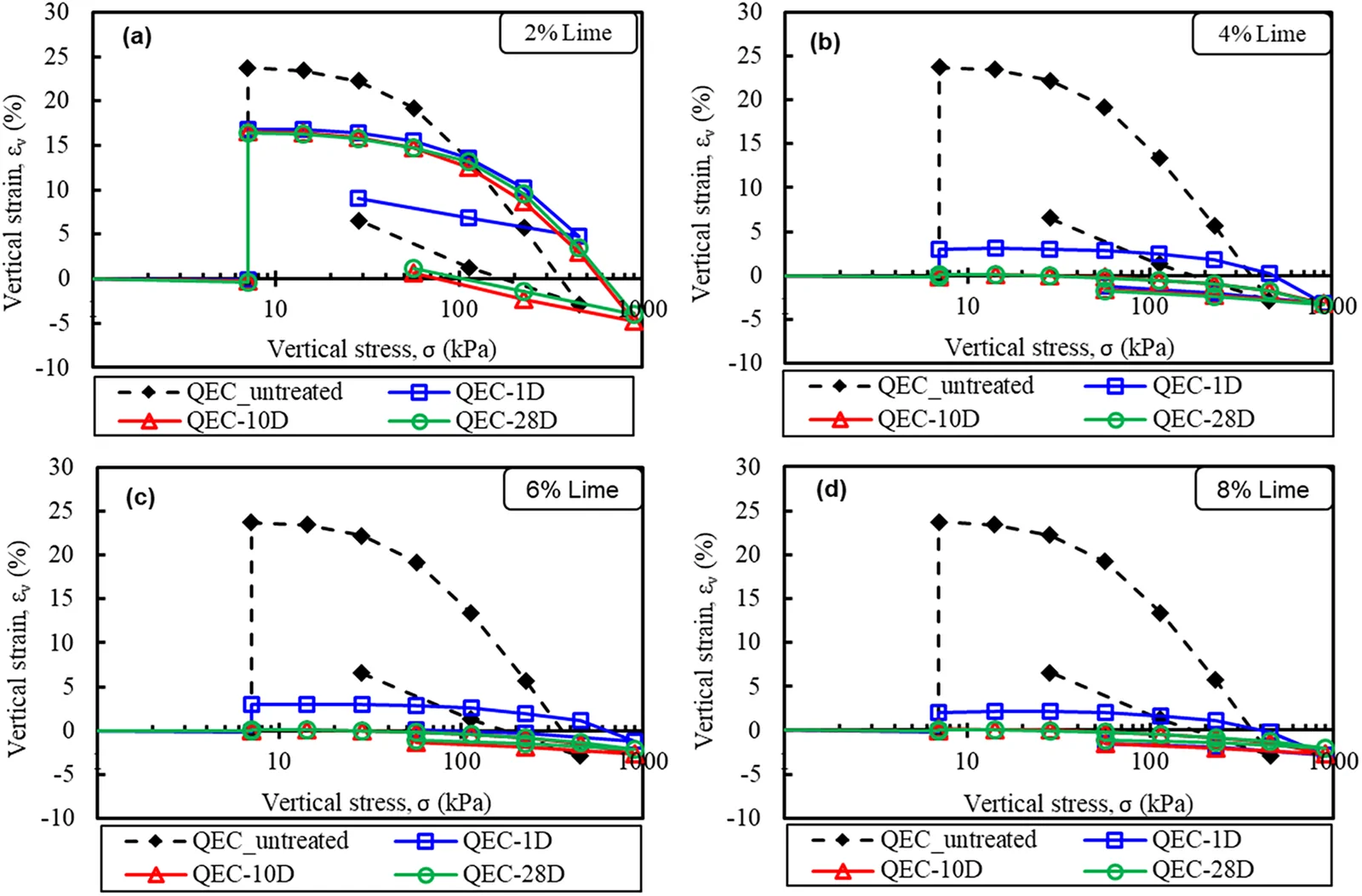
Consolidation curves of QEC under all curing days (a) at 2% lime, (b) at 4% lime, (c) at 6% lime and (d) at 8% lime.

**Fig 10:**
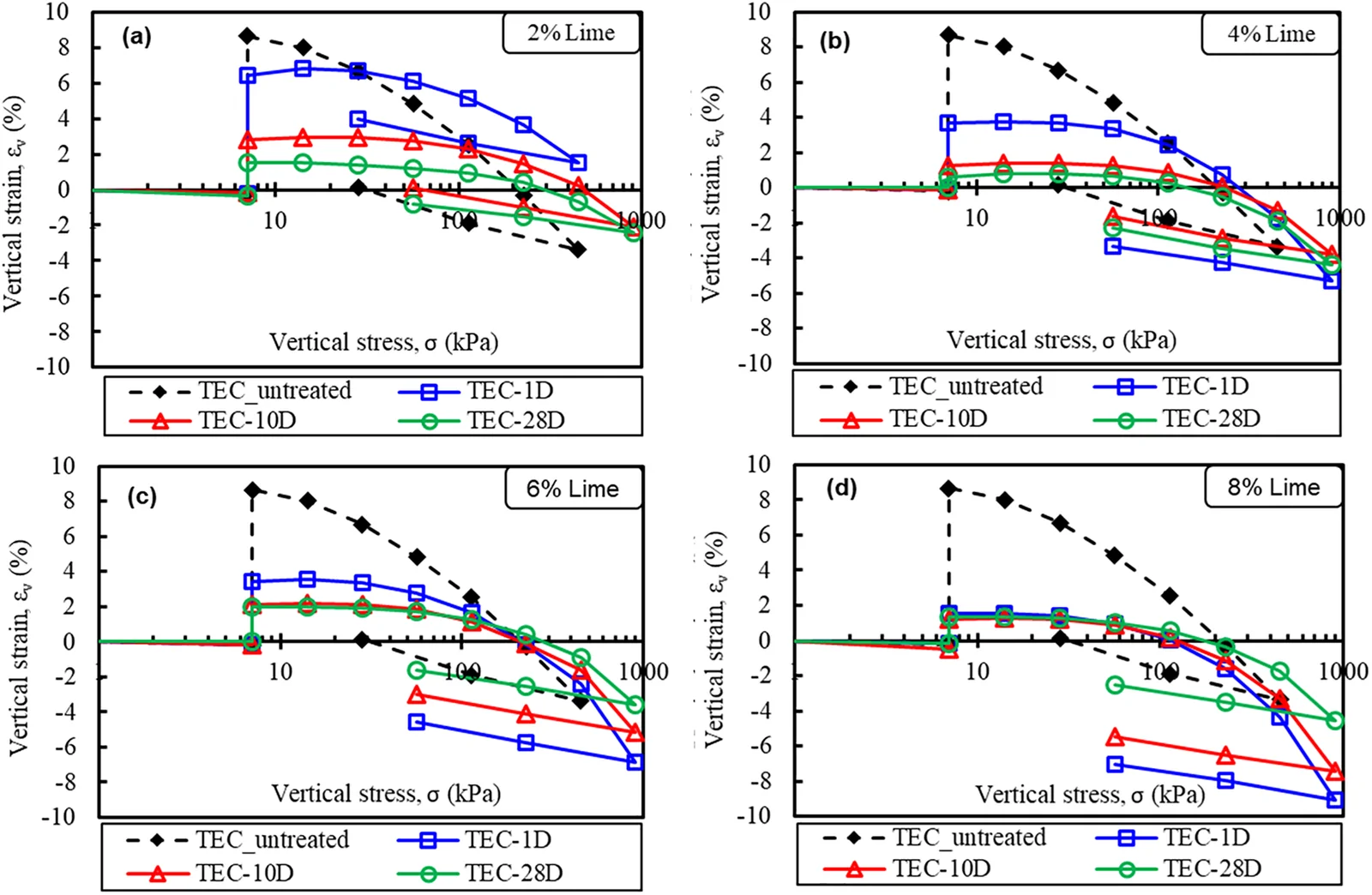
Consolidation curves of TEC under all curing days (a) at 2% lime, (b) at 4% lime, (c) at 6% lime, and (d) at 8% lime.

**Fig 11:**
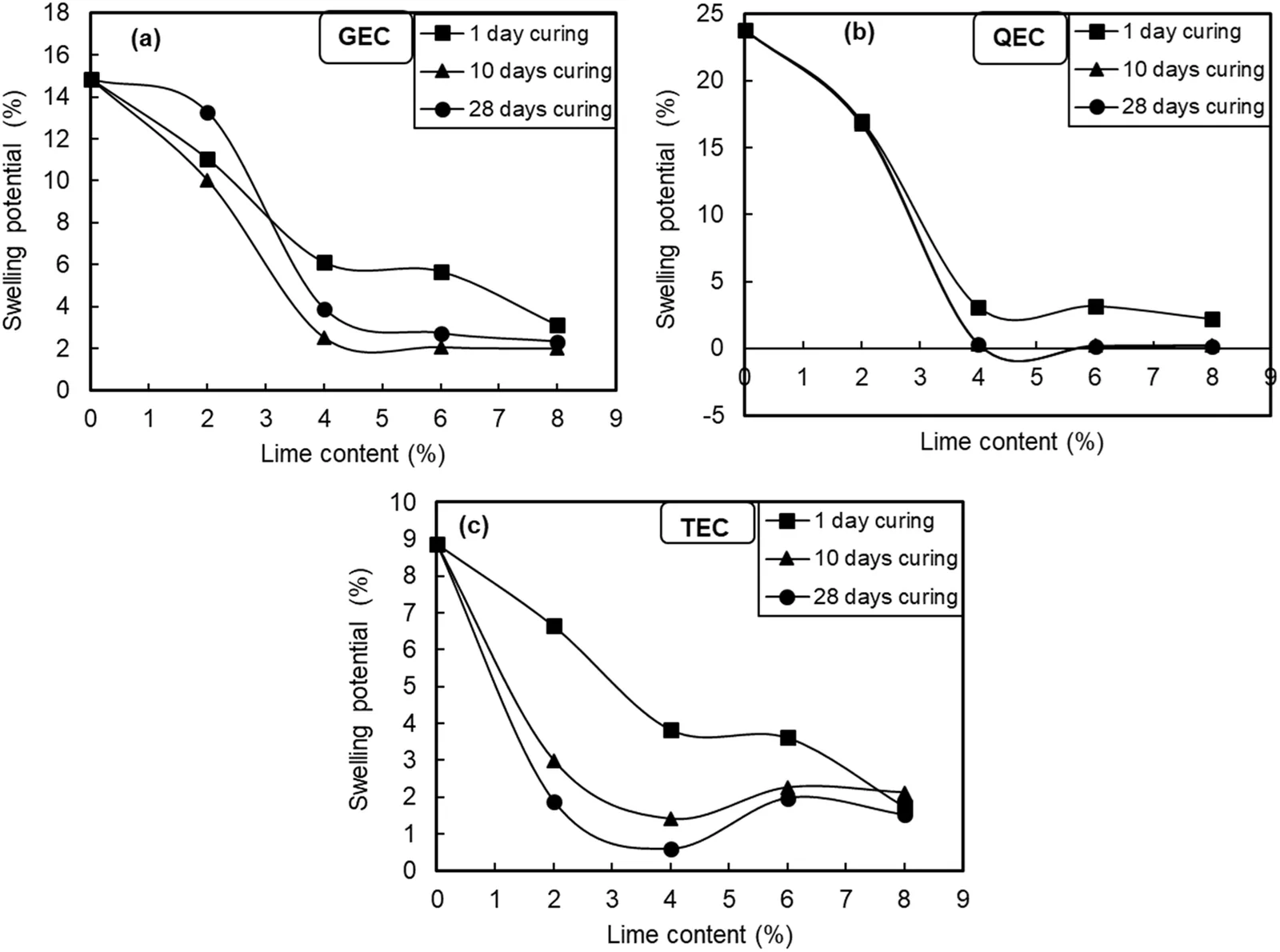
The swelling potential versus lime contents at all curing days for all types of clay (a) GEC, (b) QEC, and (c) TEC.

**Fig 12:**
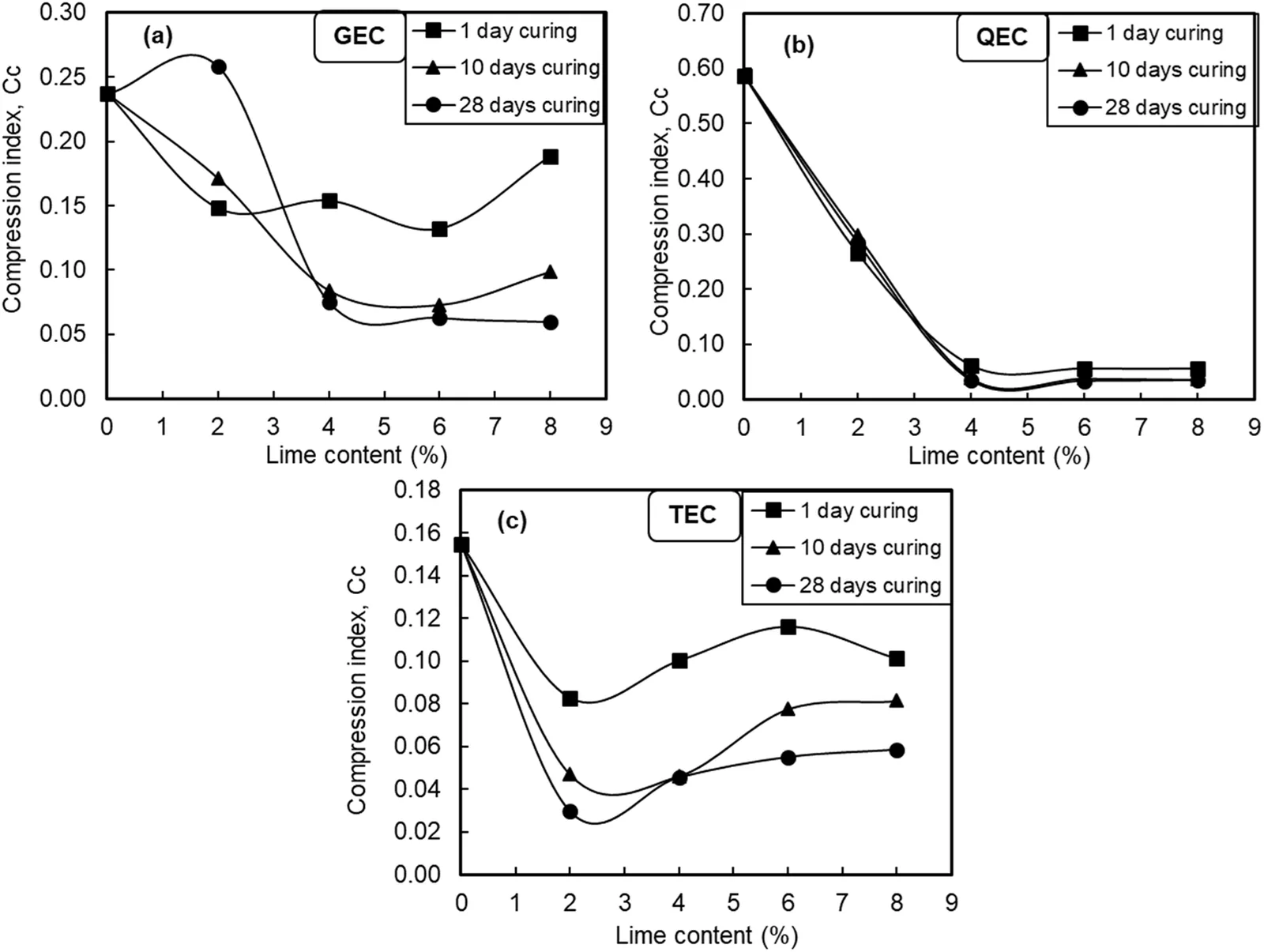
Compression index Cc, versus lime contents at all curing days for all types of clay (a) GEC, (b) QEC, and (c) TEC.

**Fig 13:**
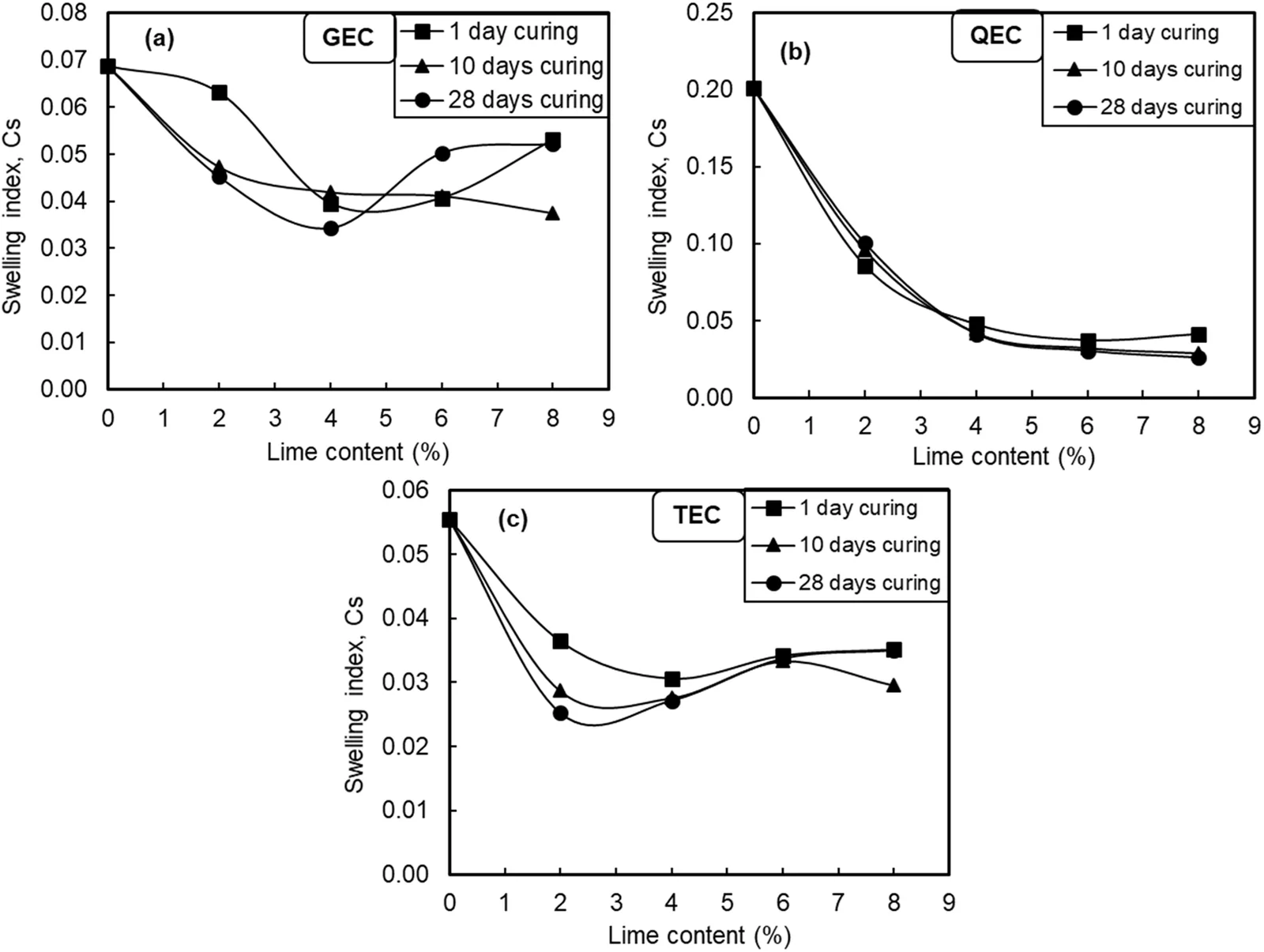
Swell index Cs, versus lime contents at all curing days for all types of clay (a) GEC, (b) QEC, and (c) TEC.

### 4.4 Soil Suction

Fig 14 presents suction pressure, versus lime contents at all curing days for all types of clay (a) GEC, (b) QEC, and (c) TEC. The suction generally varied from as low as 500 kPa to more than 11000 kPa.

**Fig 14:**
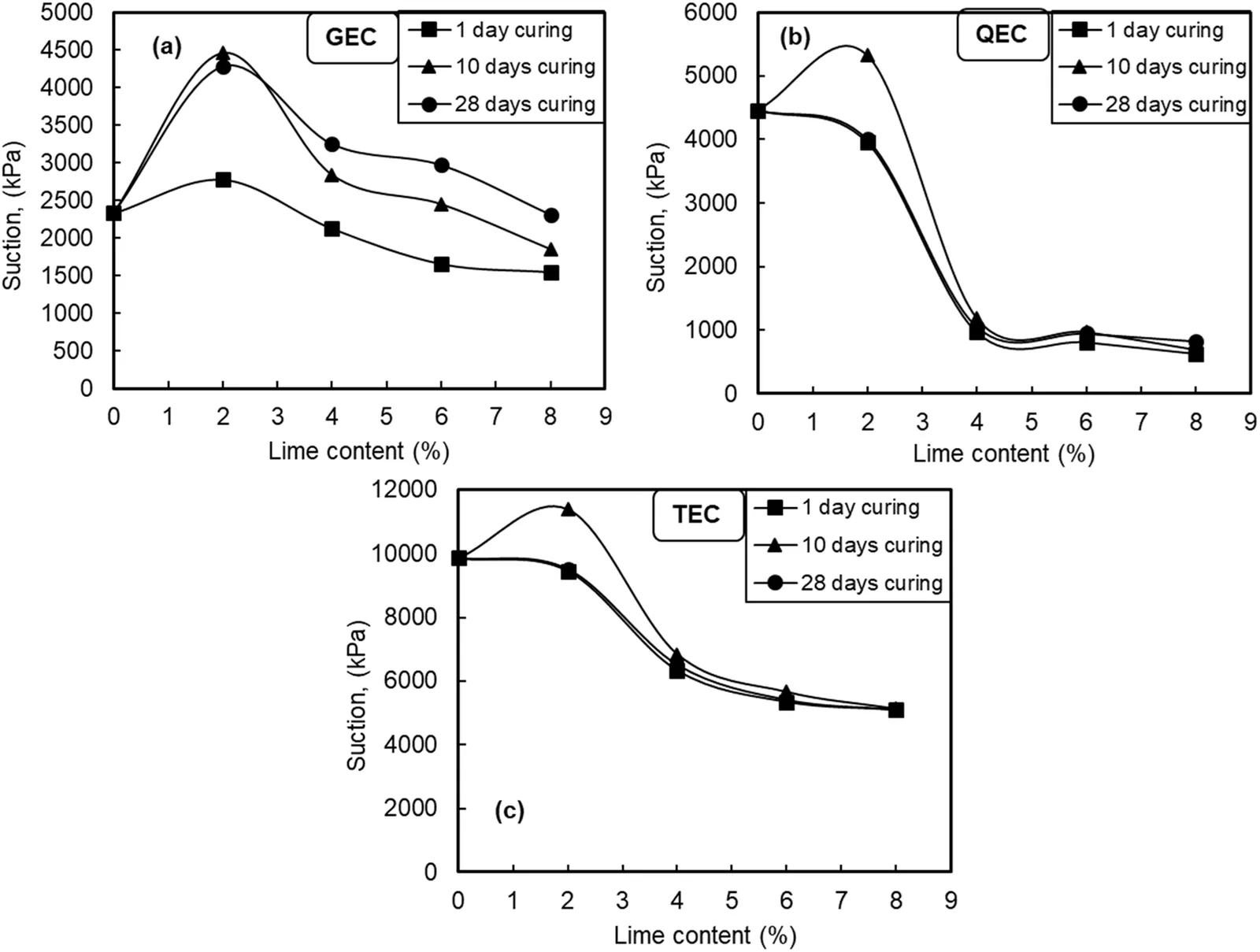
Suction pressure versus lime contents at all curing days for all types of clay (a) GEC, (b) QEC, and (c) TEC.

## 5 Discussion

The addition of lime to the compacted expansive clay has been found to have positive effects, enhancing the strength and providing stabilization to expansive soils with regard to excessive volume changes on wetting and drying. Lime treatment has been shown to decrease the plasticity index property values indicating a reduction in the plasticity (PI) [61–63]. This reduction in PI is not significant beyond a lime content of 6% [64]. Excessive lime addition can reduce the workability of the clay, hinder compaction, and possibly weaken the bonds between particles. Examining the pH values for the clays mixed with different lime contents indicated that an alkaline environment is reached rapidly. The initial lime consumption point (alkalinity >12) is satisfied at low dosages (2–4%) for all tested soils. Differences among GEC, QEC, and TEC are minor; TEC tends to have slightly higher pH at each lime content while QEC is marginally lower, implying higher clay activity.

The increase in lime content leads to a decrease in the maximum dry density (MDD) and an increase in the optimum moisture content (OMC) as shown in Fig 7. All three soils show higher **OMC** as lime content increases, reflecting the additional water required for the lime hydration and pozzolanic reactions. These changes in compaction parameters can be attributed to the lower specific gravity of lime, the aggregation of expansive clay particles, and the increased void ratio [65]. The obtained trend of the MDD and OMC parameters in this study is compatible with the results of other studies, such as [65–67].

Lime has emerged as a powerful technique to face the challenges posed by expansive soils. Firstly, lime affects the nature of expansive soils about their affinity to water. When these soils come in contact with moisture, they promptly absorb it, causing them to swell and inflate, jeopardizing the stability of any structures built upon them. Lime, however, acts as a flocculating agent, causing clay particles to clump together instead of dispersing and attracting water. This reduces the overall surface area available for water absorption, effectively curbing the swelling potential.

Beyond immediate modification, lime stabilization triggers long-term pozzolanic reactions between the lime and clay minerals, forming cementitious compounds that significantly enhance cohesion and shear strength. These chemical shifts reduce soil plasticity and stickiness, improving workability and resistance to deformation during construction. Furthermore, lime alters the soil fabric to create a more open pore structure; this improves internal drainage and mitigates the swelling risks associated with seasonal moisture fluctuations. While the success of this technique remains dependent on soil mineralogy, lime dosage, and curing conditions, it continues to be a cost-effective and reliable stabilization method for infrastructure projects.

The results are presented in terms of vertical strain versus vertical stress on a logarithmic scale (strain-log σ), as shown earlier in Figs 8-10 for the expansive clays used in this study for both conditions, with lime and without lime. It can be observed that for all clays tested, the consolidation curves are nearly parallel, especially at the high lime contents. The compressibility of Al-Ghatt and Tabuk clays, as shown in Figs 8 and 10, is primarily controlled by the shearing resistance at near-contact points, and volume changes occur by shear displacement and/or sliding between particles. In contrast, the long-range electrical repulsive forces that come from densely adsorbed ions, dissociated surface groups, or ions that have been replaced within the crystal structure are primarily responsible for the compressibility of Al-Qatif clay (Fig 9). For swelling clays, similar findings were also documented by [68].

Fig 11 (a, b, c) presents the variation of swelling potential (SP), for untreated and treated clays tested. It’s clear that the addition of lime to expansive clays experienced a reduction in swelling potential because of the lime addition. The figures show that adding 6% lime and increasing curing time reduces swelling and enhances consolidation for all three clays, but with different magnitudes of improvement. The reduction in swelling potential was observed to be more significant for QEC than for GEC and TEC. This is attributed to lime addition being more effective for soils with high montmorillonite content (as for QEC). Similar results were reported by [69–72].

On the other hand, Figs 12-13 (a, b, c) present the variation of compression index (C_C_), and swell index (C_S_) for untreated and treated clays tested, respectively. The descending trend for these variables can be observed up to 4% lime, and then an ascending trend (with a different extent) is recovered during the curing periods for natural soil. The variation is less for treated specimens with more than 4% lime. This is because more cations are removed from the clay matrix by calcium ions when the lime dose is raised over 4%, resulting in more noticeable flocculation reactions. The final outcome is a dense flocculated structure with a relatively higher concentration of crystallized calcium ions. When the added lime is greater than 4%, the excess lime added releases the silica and alumina phases from the clay particle surfaces, which bridge the clay particles due to the formation of relatively strong pozzolanic bonds. The excess lime added then simply crystallizes. Multiple researchers have demonstrated similar observation results [70,73,74].

Ghobadi et al (2014) [75] studied the effect of pH value on the shear strength parameters of clays and found that the values of undrained cohesion and friction angle increased with the increase in the pH values of the pore fluid, achieving highest values at a pH value of 9. The stabilizing impact of alkalinity on clay soils is very effective. The addition of lime to the Saudi clays selected from Al-Ghat, Al-Qatif, and Tabuk drove the state of alkalinity from nearly neutral to pH values exceeding 12.

The response of the three naturals clays to hydrated lime additives is found variable in magnitude. This is dependent on the clay mineralogy, pH value, density, and the state of exposure to weather conditions. It was observed that Al-Qatif clays require at least 3–4% to bring down the swell ability down by 90% while only 2–3% lime was found to be enough for Tabuk clay. Al-Ghatt clay required more than 4% to reduce the swelling potential by 90%.

The curing time is important to achieve satisfactory hydration 28 days is seen as suitable to judge the strength and compressibility parameters and was found to give the lowest swell potential for all clays investigated in this study. Due to the time dependence of soil lime reactions, for varying curing times, the cation exchange reactions which occur between the calcium cations of the lime and ions like potassium or sodium attached to the surfaces of the clay particles can result in altering the density and consistency, which remain changing until hydration is complete. Moghal and Elkady (2012) [55] subjected the lime-treated expansive clay at three curing times to water flow and found that leachability is reduced with increased curing time.

The compressibility and volume change profiles given for the three clays can serve as a good guide to select the percentage of lime additives to be used for a particular application or project.

We recommend considering leachability that can take place in extended periods for a better long-term performance. Moghal et al. (2015) [68] stated that the ability of the clay under severe leaching circumstances, the soil’s ability to hold the stabilizer (lime) is high when the measured pH value of the leaching solution indicates anacidic orlow-alkalinity medium. It is suggested to add extra amount of lime to achieve better stability over a longer period.

The suction of the tested clay samples taken from the three areas tends to be higher with the addition of asmall amount of lime but reduces with further addition of lime. Mixtures with a small amount of lime 1% to 2% indicated high suction levels compared to the untreated clays, as shown in Fig 14.

The long-term durability of lime-stabilized soils is still a major concern in geotechnical practice, even if this study shows notable short-term benefits. The stability of the cementitious pozollonic compounds determines how long stabilization will last. However, the system’s pH can be lowered by processes like carbonation, which is the reaction of atmospheric carbon dioxide with lime to generate calcium carbonate. This could destabilize these bonds and eventually cause strength loss. Furthermore, hydraulic and mechanical integrity can be seriously impacted by environmental conditions, such as leaching and cyclic wetting-drying. By substituting calcium cations, extended leaching may undo the positive benefits of cation exchange, increase plasticity and swell pressure during several years of service.

As a result, the dosage thresholds found in this study (2–4%) guarantee instantaneous swell reduction; however, in areas with significant groundwater changes, larger lime levels would be required to reduce leaching.

Examining Table 4, the influence of the mineralogical composition on lime stabilized soils is significant. Bell,1990 [25] stated that by the addition of lime, the plasticity of montmorillonite was reduced whilst that of kaolinite and quartz was increased. Compared to kaolinite-dominant soils (TEC in this study; UK kaolinite in Bell, 1996), which stabilize well at 2–4%, soils dominated by smectite or montmorillonite (QEC in this study) consistently require higher lime dosages to achieve equivalent swell reductions. This is explained by the fact that smectite minerals have a far higher specific surface area and cation exchange capacity (800 m²/g) than kaolinite (15 m²/g), which requires more Ca2 + ions to fully saturate the exchange complex. The work of Abduljauwad (1991) [3] yielded higher lime content needed, and this is attributed to the use of commercial unidentified lime material. Bouras et al. (2020) and Basma & Tuncer (1991) [33,37]. The testing methodology was found to have influence, as some studies employ free swell index methods. These methods can reveal higher reduction percentages than those based on oedometer swell potential under surcharge, because the free swell test applies no restricting stress. The influence of curing temperature and duration is also found significant, and Bouras et al. (2020) [33] showed that swell reduction exceeded 90% when curing was extended from 28 to 90 days at 20°C. Similar to the current work, Bell (1990) [25] employed conventional laboratory settings (20°C). Differences can arise from specimens compacted at different energies (standard vs. modified Proctor) or at wet of optimum versus dry of optimum conditions. Different initial fabric and void ratios directly influence the measured swell potential and the apparent effectiveness of lime. The works of Sendilvadivelu et al. (2025) [76] is presented to show that other additives along with lime can enhance the stability and improve the strength.

**Table 4:** Lime stabilization previous works compared to the current study.

Study	Location	Dominant Minerals	USCS	Lime %	Curing Period	Test Method	Swell reduction%	Key Remarks
Present study – TEC	Tabuk, Saudi Arabia	Kaolinite, mica	CL	2-3	1, 10, 28	1D Oedometer	>90%	Lowest lime demand
Present study – QEC	Al-Qatif, Saudi Arabia	Illite, palygorskite	CH	3-4	1, 10, 28	1D Oedometer	>90%	higher lime needed due to large specific surface area
Present study – GEC	Al-Ghatt, Saudi Arabia	Kaolinite, dominant	CL	>4	1, 10, 28	1D Oedometer	>90%	Despite kaolinite dominance, specific mineral matrix requires higher dosage than TEC
Abduljauwad (1991) [3]	Al-Qatif, Saudi Arabia	Montmorillonite, illite	CH	4-8	1, 7, 28	1D Oedometer	100% (at 8% lime)	Commercial lime for preconstruction treatment of Al-Qatif clays
Bell (1991) [25]	United Kingdom	Kaolinite; Montmorillonite	CL-CH	4-6	7, 28	1D Oedometer	100% (at 6% lime)	
Bouras et al. (2020) [33]	France	Smectite	CH	3-6	7, 28, 90	1D Oedometer	87% (at 4% lime)	Lime showed 87% swell decrease
Basma and Tuncer (1991) [37]	Jordan	Montmorillonite, kaolinite	CH	2-8	2, 7,28	1D Oedometer	70–90 (at 3–6%)	Swell reduction proportional to lime content; accelerated by longer curing
Sendilvadivelu et al. (2025) [76]	India	Illite, kaolinite and Quart	CL/CH	4.5	0, 7,14,28	UCS	–	Lime is enhanced by other additive. Reduced plasticity by 69% and increased compressive strength.

### 5.1 Scope and limitations

While this research provides essential baseline data on the mechanical and physical improvement of lime-treated expansive clays under standard laboratory conditions (T = 20°C ± 2°C, RH = 95%), certain limitations apply to the direct extrapolation of these findings to field conditions. Specifically, the high in-situ surface temperatures characteristic of arid regions like Saudi Arabia, which often exceed 50°C, are known to significantly accelerate pozzolanic reaction kinetics, potentially leading to faster early-strength development than reported herein. Furthermore, long-term durability aspects, such as wetting–drying cycles and chemical leaching under multi-year field service, were outside the immediate scope of this fundamental phase. Future research will focus on evaluating these environmental durability factors under simulated arid climate cycles.

## 6 Conclusions

This study investigated three types of expansive soils known to be problematic for construction in the Kingdom of Saudi Arabia. Al-Ghatt clay, Al-Qatif clay, and Tabuk clay were investigated for compressibility and swell for variable hydrated lime additives and compared to untreated clays. The relevant geotechnical parameters were studied and presented. The role of hydrated lime addition was quantified, and a recommendation for reducing the swell potential by 90% is given for the three investigated soils. The following statements can be made:

- The mineralogy and chemistry of the expansive soil determine the dose of hydrated lime. The required lime content to achieve a 90% reduction in swell potential is directly governed by clay mineralogy. Tabuk clay (Kaolinite-dominant) requires the lowest dosage at 2–3%, while Al-Qatif clay (Smectite-dominant) requires 3–4%. For Al-Gatt clay, despite lower initial activity, it necessitates **>**4% lime to reach the same 90% reduction threshold due to its specific mineral matrix.
- The curing time is a significant factor that controls the hydration process and facilitates the pozzolanic reactions. The 28-day curing period was identified as the optimal duration for stabilizing hydro-mechanical properties. Total suction values increased significantly at low lime dosages (**1–2%**) due to initial hydration but stabilized or decreased at higher dosages (**>6%**) as the soil fabric transformed into a more open-pore, granular structure.
- Increased lime content reduces the suction and consequently lessens the water absorption.
- The study recommends considering leachability when designing for long-term performance.

When a modest amount of lime is added, the suction of clay tends to be higher, but when more lime is added, the suction decreases. This behavior is crucial for engineering applications, where the use of lime-stabilized soils can help prevent excessive volume change.

## Abbreviation

CH: Clay with high plasticity
CL: Clay with low plasticity
GEC: AL-Ghatt expansive clay
QEC: AL-Qatif expansive clay
TEC: Tabuk expansive clay
SP: Swelling potential
CC: Compression index
CS: Swell index
